# A Bifunctional Electrocatalyst for OER and ORR based on a Cobalt(II) Triazole Pyridine Bis‐[Cobalt(III) Corrole] Complex

**DOI:** 10.1002/anie.202302208

**Published:** 2023-04-13

**Authors:** Abdalaziz Aljabour, Houssein Awada, Luyang Song, He Sun, Simon Offenthaler, Farzaneh Yari, Matthias Bechmann, Markus Clark Scharber, Wolfgang Schöfberger

**Affiliations:** ^1^ Institute of Organic Chemistry Laboratory for Sustainable Chemistry and Catalysis (LSusCat) Johannes Kepler University (JKU) Altenberger Straße 69 4040 Linz Austria; ^2^ Institute of Applied Chemistry Department of Science and Technology IMC University of Applied Sciences Krems Wien Piaristengasse 1 3500 Krems Austria; ^3^ Institute of Physical Chemistry and Linz Institute of Organic Solar Cells Johannes Kepler University Linz Altenberger Straße 69 4040 Linz Austria

**Keywords:** Cobalt, Corroles, Electrochemistry, Oxygen Evolution, Oxygen Reduction

## Abstract

As alternative energy sources are essential to reach a climate‐neutral economy, hydrogen peroxide (H_2_O_2_) as futuristic energy carrier gains enormous awareness. However, seeking for stable and electrochemically selective H_2_O_2_ ORR electrocatalyst is yet a challenge, making the design of—ideally—bifunctional catalysts extremely important and outmost of interest. In this study, we explore the application of a trimetallic cobalt(II) triazole pyridine bis‐[cobalt(III) corrole] complex Co^II^TP[Co^III^C]_2_ **3** in OER and ORR catalysis due to its remarkable physicochemical properties, fast charge transfer kinetics, electrochemical reversibility, and durability. With nearly 100 % selective catalytic activity towards the two‐electron transfer generated H_2_O_2_, an ORR onset potential of 0.8 V vs RHE and a cycling stability of 50 000 cycles are detected. Similarly, promising results are obtained when applied in OER catalysis. A relatively low overpotential at 10 mA cm^−2^ of 412 mV, Faraday efficiency 98 % for oxygen, an outstanding Tafel slope of 64 mV dec^−1^ combined with superior stability.

## Graphical abstract

By designing the structure of the catalyst, possessing Co^II^ and Co^III^ reaction centers, we achieve nearly 100 % selective activity during ORR to H_2_O_2_. Similarly, promising results are obtained when applied in OER catalysis. A relatively low overpotential at 10 mA cm^−2^ of 412 mV, a Faraday efficiency of 98 % for oxygen evolution, and an outstanding Tafel slope of 64 mV dec^−1^ are combined with a superior stability.

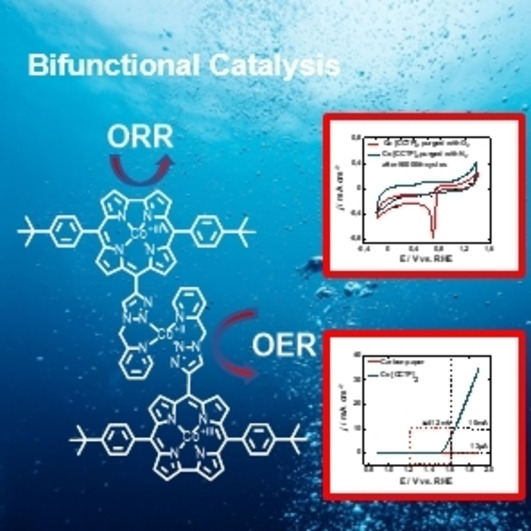

The depletion of traditional fossil fuels requires a severe revision of today's energy generation.^[1]^ As an auxiliary approach, sustainable and clean electrocatalytic technologies promote the realization of the renewable energy generation positively to relieve industries from fossil sources.^[2]^ Especially, the oxygen evolution reaction (OER) and the oxygen reduction reaction (ORR) are two key processes in the fuel cell applications, being attractive and promising alternatives.^[3]^ To achieve efficient, low‐cost, selective, and stable electrosynthesis, the design of electrocatalysts is, however, of huge importance and a considerable technological challenge yet.^[4]^ To date, mainly precious noble metals, alloys, carbon‐based materials but also incorporated hybrid systems such as doping with heteroatoms or metals remained impractical since the scarcity and complexity of these materials hindered their wide application and thus remained as an obstacle for their broad application at technological level.^[5]^ A variety of homogeneous and heterogeneous water‐oxidation catalysts based on transition metals have been developed, including complexes of Cu, Mn, Ru, Ir, and Co.[^4^, ^6^] All of these catalyst systems are proposed to proceed through a high‐valent intermediate.^[6]^ Corroles are trianionic ligands known to stabilize metal ions in their high‐valent oxidation states.^[7]^ Nocera and co‐workers reported a hangman cobalt corrole for the efficient oxidation of water.^[7b]^ During the oxygen‐evolution reaction, the corrole macrocycle stabilizes the center cobalt ion as Co^IV^ and the corrole macrocycle, in contrast to the closely related porphyrin‐based systems, tends to be involved as a non‐innocent ligand, forming π‐radical cation species.

Cobalt corrole complexes are also reported for the electron oxygen reduction reactions.^[8]^ The ORR is sophisticated since the oxygen reduction can occur via either two or four electron pathways, yielding in hydrogen peroxide or water as valuable products, respectively, thus making it complex to direct the product selectively.^[9]^

Cobalt(III) corroles are known for their intrinsic preference of catalyzing the two‐electron reduction of oxygen to H_2_O_2_ due to their high d‐electron counts, suppressing the 4 e^−^ reduction.^[10]^ The two‐electron reduction product, H_2_O_2_, is an immensely used, as green oxidizing agent with broad application in chemical industry (i.e., in organic synthesis, paper and pulp processing, as bleaching agent), wastewater treatment, or serves as a potential energy carrier competitive to compressed to H_2_ gas.[^9b^, ^10a^] Although hydrogen peroxide is environmental friendly itself because of the decomposition products being water and/or oxygen, the current production over the anthraquinone oxidation method thereof is hazardous, energy‐intensive and waste demanding.[^9a^, ^10b^] Therefore, the electrochemical two‐electron ORR process provides a highly attractive alternative synthesis route.^[11]^

Consequently, the development of efficient bifunctional catalysts has lead to a fast evolving research area.[^3b^, ^12^] Although there are some reports of bifunctional catalysts for the reversible conversion of protons into H_2_, reports on nonprecious metal‐based electrocatalysts, which can efficiently oxidize water to oxygen and in addition reduce oxygen are rare.^[13]^ Such catalysts are in high demand. In the underlying work, we present a novel bifunctional electrocatalyst containing one cobalt(+II) and two cobalt(+III) complex centers (Figure 1). We have covalently embedded a cobalt(+II) triazole pyridine complex (capable of performing water oxidation to O_2_ and H^+^),^[14]^ between two cobalt(III) corrole complexes catalyzing the two‐electron reduction reaction of oxygen to H_2_O_2_. The reaction protocol involved the synthesis of the cobalt(III) 5,15‐4‐*t*‐butylphenyl‐10‐triisopropylsilylethynyl corrole **1** (Scheme 1).^[15]^

**Figure 1:**
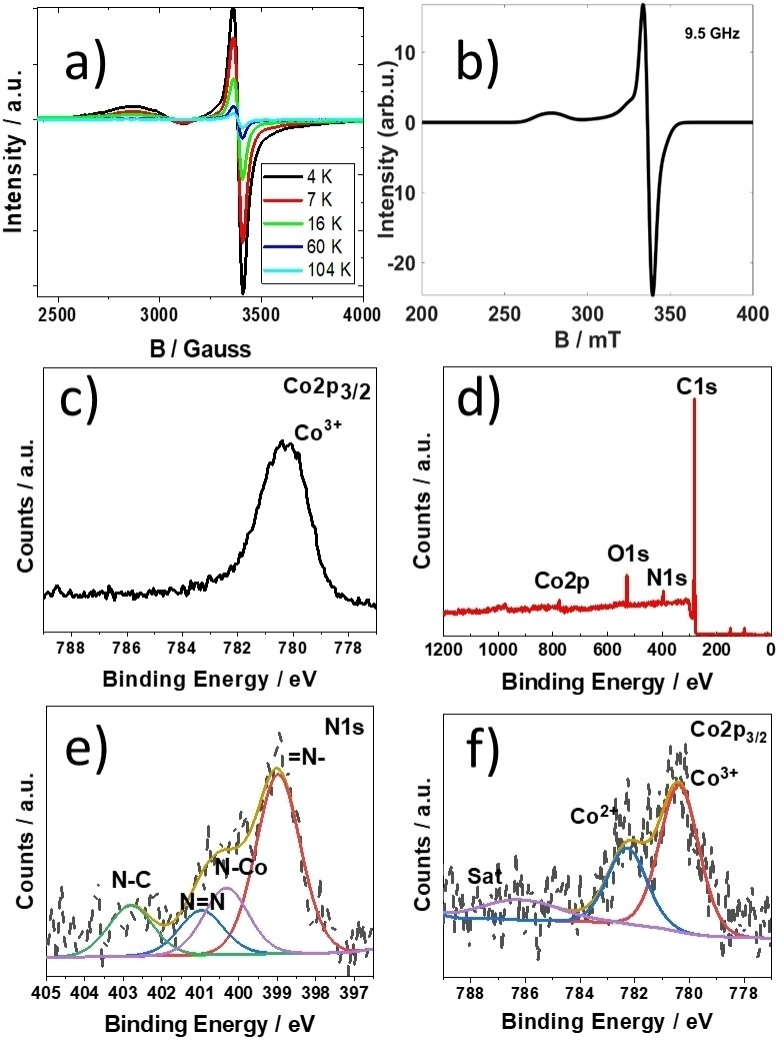
a) Series of EPR spectra for a polycrystalline sample of **3** plotted to show the temperature dependence of the EPR signal. b) simulated solid state EPR spectrum c) XPS survey scan of Co^II^TP(Co^III^C)_2_](OH)_2_ **3** on carbon paper. d) XPS narrow scans for Cobalt (Co2p_3/2_) present in the precursor **2**. XPS narrow scans for e) Nitrogen (N1s) and f) Cobalt (Co2p_3/2_) present in Co^II^TP[Co^III^C]_2_ **3** on carbon paper.

**Scheme 1:**
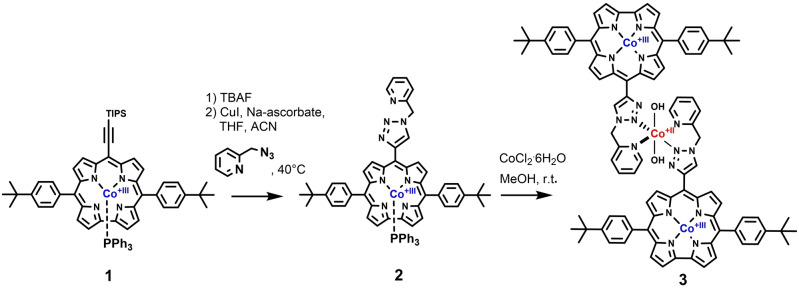
Synthesis of the bifunctional [Co^II^TP(Co^III^C)_2_](OH)_2_ catalyst **3** for oxygen evolution reaction (OER) at the cobalt(II) center and oxygen reduction reaction (ORR) at the two cobalt(III) centers of the trimetallic complex.

After desilylation of the cobalt(III) corrole with TBAF, azide‐alkyne Huisgen cycloaddition with 2‐(azidomethyl)‐pyridine, CuI, and Hunig base, in MeCN/THF (1 : 1) was performed to obtain the diamagnetic cobalt(III) 5,15‐4‐*t*‐butylphenyl‐10‐{4‐[‐(2‐methyl‐pyridine)‐1,2,3‐triazole]} corrole precursor **2** in excellent yield.

Subsequent complexation with CoCl_2_⋅6 H_2_O afforded the paramagnetic cobalt(II) 10‐{4‐[‐(2‐methyl‐pyridine)‐1,2,3‐triazole]} [cobalt(III) 5,15‐4‐*t*‐butylphenyl‐corrole]_2_ complex [Co^II^TP(Co^III^C)_2_](OH)_2_ **3**.

Due to the molar magnetic susceptibility tensor anisotropy Δχ the ^1^H NMR spectrum of [Co^II^TP(Co^III^C)_2_](OH)_2_ complex **3** in CDCl_3_ (Figure S15) exhibits large pseudocontact chemical shift of each nucleus and therefore a complete assignment of the resonances is impossible. MALDI‐TOF mass spectrum however, exhibits the molar peak [*M*+H]^+^ for Co^II^TP[Co^III^C]_2_ **3** at *m*/*z*=1612.519 and [*M*+H]^+^ for Co^II^TP(H_2_O)[Co^III^C]_2_ and at *m*/*z*=1631.481 (Figure S18).

Variable temperature powder EPR spectra of Co^II^TP[Co^III^C]_2_ **3** were recorded at 104, 60, 16, 7 and 4 K (Figure 1a). The signal amplitude increases as the temperature decreases, suggestive of a doublet and quartet ground state. The EPR spectrum at 4 K suggests for the existence of two paramagnetic Co species (low‐spin d^7^ Co^II^ (I=1/2) and high‐spin d^7^ Co^II^ (I=3/2)) with axial g‐factors (g_⊥_=2.01, g‖=2.03) and (g_⊥_=2.01, g‖=2.41). The resulting simulated spectrum is illustrated in Figure 1b. Detailed investigations are in progress and beyond the scope of the underlying work.

As a reference, Figure 1c shows the narrow XPS scan for Co2p_3/2_ of the Co^III^ triazol pyridine corrole **2**. The signal exhibits a shift value of 780.4 eV.

Figure 1d shows the broad region of XPS survey scan spectra of the [Co^II^TP(Co^III^C)_2_](OH)_2_ **3** on carbon paper before the electrocatalysis reactions. The atomic percentages of C, N, O, and Co are provided in Supporting Information Table S1. Figure 1c also indicates that the [Co^II^TP(Co^III^C)_2_](OH)_2_ **3** is adsorbed on the electrode surface. The nitrogen N1s peak at 398.98 eV is related to =N− bond in corrole macrocycle after cobalt metalation (Figure 1e).^[16]^ The peak located at 400.33 eV stems from the contribution from central N−Co bond.^[17]^ Two distinct peaks were observed at 400.98 (N=N) and 402.81 eV (N−C), which can be assigned to the triazole group formed after via click reaction.^[18]^ The narrow scan of Co2p_3/2_ spectrum (Figure 1f) shows several peaks in the range of 775 eV to 789 eV. Specifically, the peak at binding energy of 780.37 eV can be assigned to Co^III^ (please also refer to Figure 1c), while the peak at 782.28 eV can be assigned to the center Co^II^ ion of the [Co^II^TP(Co^III^C)_2_](OH)_2_ complex **3**. The ratio of these two peaks is precisely 1 : 2 (Co^II^/Co^III^). The peak at 786.16 eV is the satellite peak, which further corroborates the existence of a Co^II^ ion present in [Co^II^TP(Co^III^C)_2_](OH)_2_ **3**.[^17^, ^19^]

The electrochemical ORR activity of [Co^II^TP(Co^III^C)_2_](OH)_2_ **3** is first determined by conducting cycling voltammetry (CV) (Figure 2a). In a standard three electrode configuration using Pt as a counter electrode (CE), electrocatalyst **3** is loaded on glassy carbon as working electrode (WE) and Hg/HgO as reference electrode in a N_2_ and O_2_ saturated 0.1 M KOH (pH 13) at room temperature, respectively. Figure 2a reveals an obvious cathodic reduction peak at 0.70 V vs. reversible hydrogen electrode (RHE) clearly indicating the ORR activity, only present in oxygen‐saturated environment. The linear sweep voltammogram (LSV) in Figure 2b further confirms the outstanding ORR performance of [Co^II^TP(Co^III^C)_2_](OH)_2_ **3**, when measured on the rotating ring disc electrode (RRDE), with an onset potential (*E*_onset_) of 0.8 V vs. RHE (−0.1 mA cm^−2^) and a half‐wave potential (*E*_1/2_) of 0.72 V vs. RHE (−1.5 mA cm^−2^).

**Figure 2:**
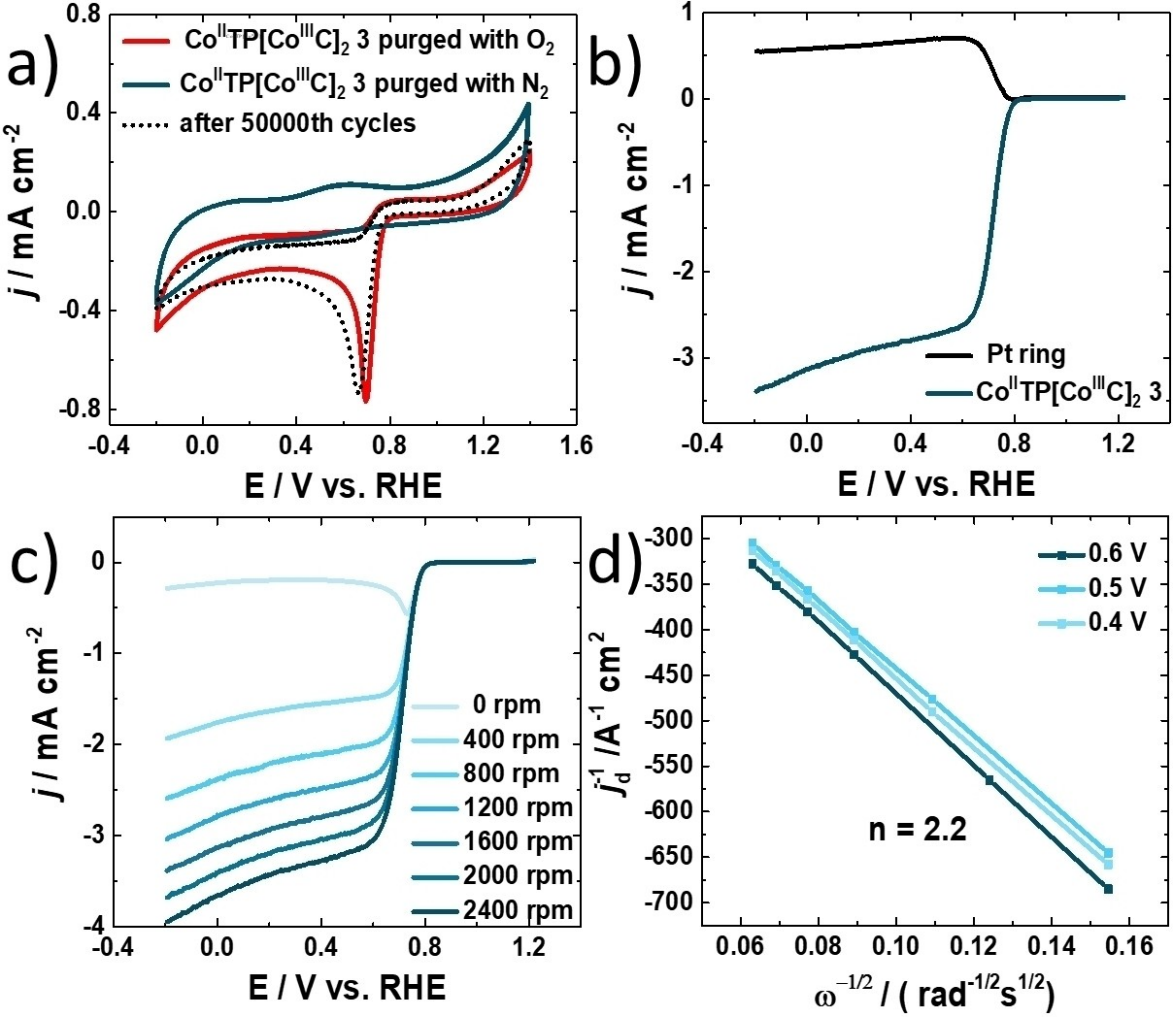
Electrochemical ORR catalytic performance of [Co^II^TP(Co^III^C)_2_](OH)_2_ **3**. a) CV curves of glassy carbon electrode (GCE) loaded with [Co^II^TP(Co^III^C)_2_](OH)_2_ in 0.1 M KOH statured with N_2_ and O_2_ separately at 50 mV s^−1^ and CV curves of [Co^II^TP(Co^III^C)_2_](OH)_2_ **3** electrode saturated with O_2_ after a continuous CV for 50 000 cycles. b) Linear sweep voltammograms (LSVs) of [Co^II^TP(Co^III^C)_2_](OH)_2_ **3** and Pt ring at 1600 rpm sweep rate: 10 mV s^−1^ measured on RRDE. c) LSVs of different rotating speed from 0 rpm to 2400 rpm measured on RRDE. d) Koutecky–Levich (K–L) plots of [Co^II^TP(Co^III^C)_2_](OH)_2_ **3** at different potentials vs. RHE. The lines are drawn by linearly fitting the corresponding dots.

In addition, the number of transferred electrons and the selectivity for H_2_O_2_ are calculated from LSVs measured by RRDE (Figure 2b) resulting in a two‐electron transfer and nearly 100 % selectivity for H_2_O_2_ production (see Supporting Information). The ORR charge transfer kinetics and the electron transfer number of [Co^II^TP(Co^III^C)_2_](OH)_2_ **3** are studied by using RDE and RRDE. The LSVs in Figure 2c exhibit the relation of the current density as a function of rotation speed, the current density increases linearly by increasing the rotation speed from 0 to 2400 rpm. Further, the number of transferred electrons for ORR are calculated from Koutecky–Levich (K–L) plots of [Co^II^TP(Co^III^C)_2_](OH)_2_ **3** at different potentials vs. RHE (Figure 2d) being almost in agreement with the theoretical value of *n*=2.0.

The electrochemical cell parameter as well as charge transfer resistance *R*_CT_ are determined by electrochemical impedance spectroscopy (EIS) (Figure 3a). Based on these measurements negligible cell and electrolyte resistances are figured out from Bode plot. The corresponding resistances are summarized in the Supporting Information in Tables S4.

**Figure 3:**
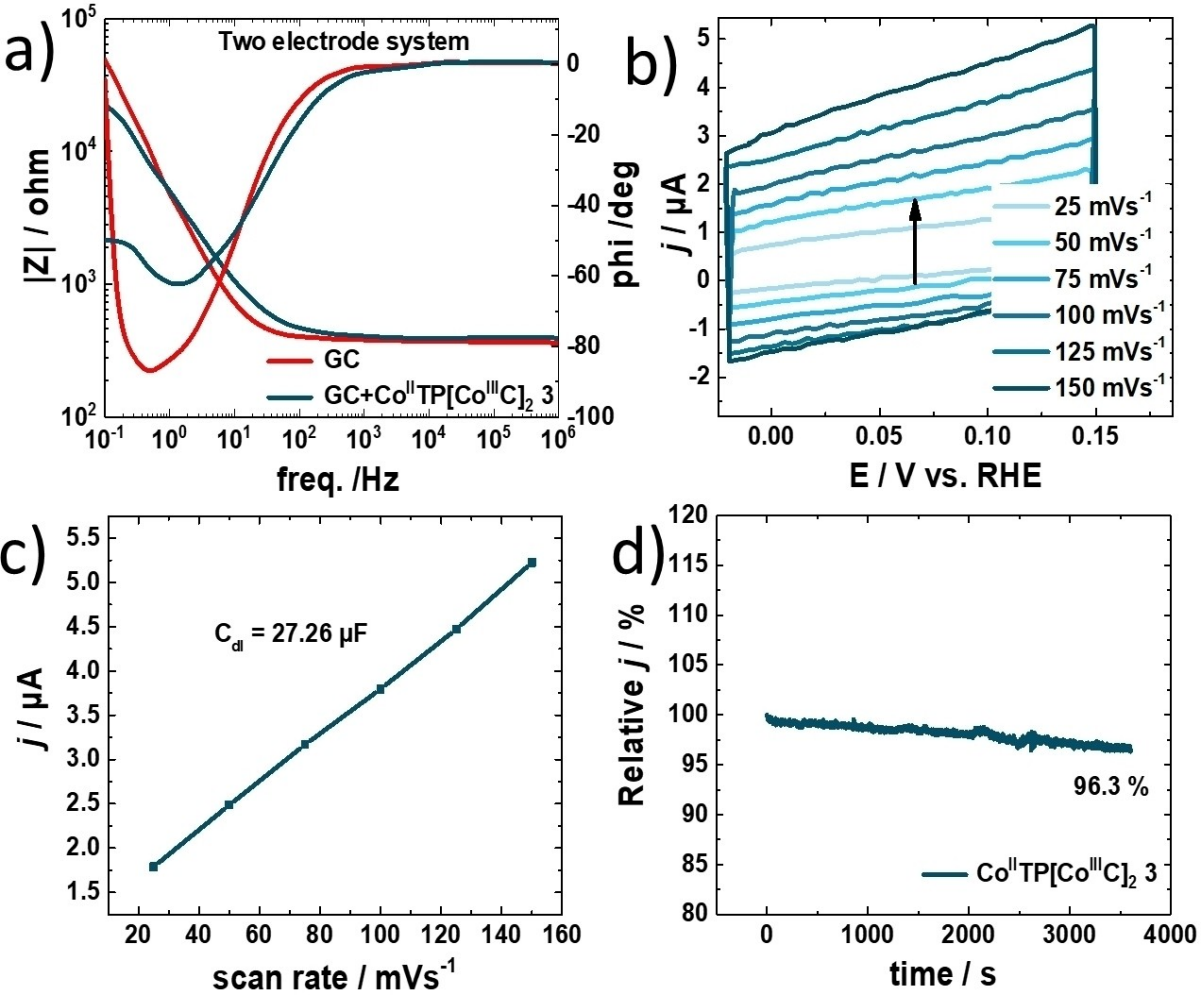
a) Bode plot recorded via electrochemical impedance spectroscopy in the frequency range of 1×10^−1^ Hz to 1×10^6^ Hz with a perturbation amplitude of 10 mV. b) Double‐layer capacitance measurements for determining electrochemically active surface area for [Co^II^TP(Co^III^C)_2_](OH)_2_ **3** catalyst in 0.1 M KOH. c) Linear dependence of the cathodic peak current versus scan rate. Double‐layer capacitance measurements for determining electrochemically active surface area for [Co^II^TP(Co^III^C)_2_](OH)_2_ **3** catalyst in 0.1 M KOH. d) I–T performance curves of [Co^II^TP(Co^III^C)_2_](OH)_2_ **3** for 3600 seconds.

The *R*_CT_ of [Co^II^TP(Co^III^C)_2_](OH)_2_ **3** during ORR is found as 913 Ω from the Bode plot. These abovementioned data explain the high catalytic activity of [Co^II^TP(Co^III^C)_2_](OH)_2_ **3** towards ORR since the lower resistance corresponds to the faster charge transfer rate in ORR. The electrochemically active surface area (ECSA) is calculated according to the electrochemical double‐layer capacitance (*C*_dl_), based on the proportional relation between the ECSA and *C*_dl_ by the equation ECSA=*C*_dl_/*C*_s_, resulting in 1.0 cm^2^ where *C*_s_ is the specific capacitance.^[9b]^ The double‐layer capacitance is determined via CV measurements in a 0.1 M KOH electrolyte in a potential range of −0.02 to 0.15 V vs RHE at increasing scan rates starting from 25 to 150 mV s^−1^ (Figure 3b). Extracted from the slope of the current density plotted as a function of scan rate, the value of the *C*_dl_ is found (Figure 3c). Besides alluring selective catalytic activity of the electrocatalyst, the stability of [Co^II^TP(Co^III^C)_2_](OH)_2_ **3** is almost equally decisive to performance. To verify the durability of [Co^II^TP(Co^III^C)_2_](OH)_2_ **3** the same electrode is characterized by chronoamperometric studies in 0.1 M KOH purged with O_2_ under static and rotating conditions. Figure 3d reveals the stability of the electrocatalyst for 3600 seconds, with only 3.7 % loss of current as compared to the initial current (Figure 3d). We associate this change to slight delamination of the active material from the electrode surface due to mechanic disturbance originating from rotating the electrode. In addition, we tested the electrode stability by CV measurements at continuous cycling for 50 000 cycles. Almost no loss of electrocatalytic ORR activity after 50 000 cycles is detected (Figure 3a, dotted black line). Note that prior to CV measurements a LSV polarization curve was recorded. Next, once the 50 000 CV cycles were finished, again LSV is done at 1600 rpm and compared to the LSV polarization curve before the CV measurements are conducted. Surprisingly, we find that the catalytic current is as good as the initial current (see Supporting Information Figure S20). The ceric sulfate titration of H_2_O_2_ reaction revealed values for TON_ORR_ and TOF_ORR_ of 2350 and 0.653 s^−1^, respectively (Figure S24).

In addition to ORR, electrocatalytic OER activity is also evaluated in 0.1 M KOH. To localize the active center of OEC within complex **3**, we have investigated bare carbon paper and complexes **2** towards OEC. Bare carbon paper and complex **2** do not show any activity towards heterogeneous OEC (Figure S22) and therefore we assume that the Co^II^ center in the pyridine triazole is the active center for OEC. Figure 4a reveals the catalytic OER activity of [Co^II^TP(Co^III^C)_2_](OH)_2_ **3** reaching the benchmark current density of 10 mA cm^−2^ at 412 mV overpotential. iR compensation and the determination of cell resistances are obtained by EIS (Figure 4b, for details on resistances see Supporting Information table S5), enabling an ideal operation of [Co^II^TP(Co^III^C)_2_](OH)_2_ **3** for OER. As proof of fast reaction kinetics, the OER Tafel slope of the bifunctional electrocatalyst is found to be 63.6 mV dec^−1^ (Figure 4c). The TON_10min_ was 15.35 and the TOF=0.026 s^−1^. Finally, the OER stability is examined by electrolysis at an overpotential of 412 mV resulting in 10 mA cm^−2^ current maintaining stable over one hour (Figure 4d). Additionally, we have thoroughly investigated the stability of complex **3** using XPS analysis before and after OEC reaction. We obtained identical XPS spectra before and after heterogeneous catalysis reaction (Figure 1f and Figure S25).

**Figure 4:**
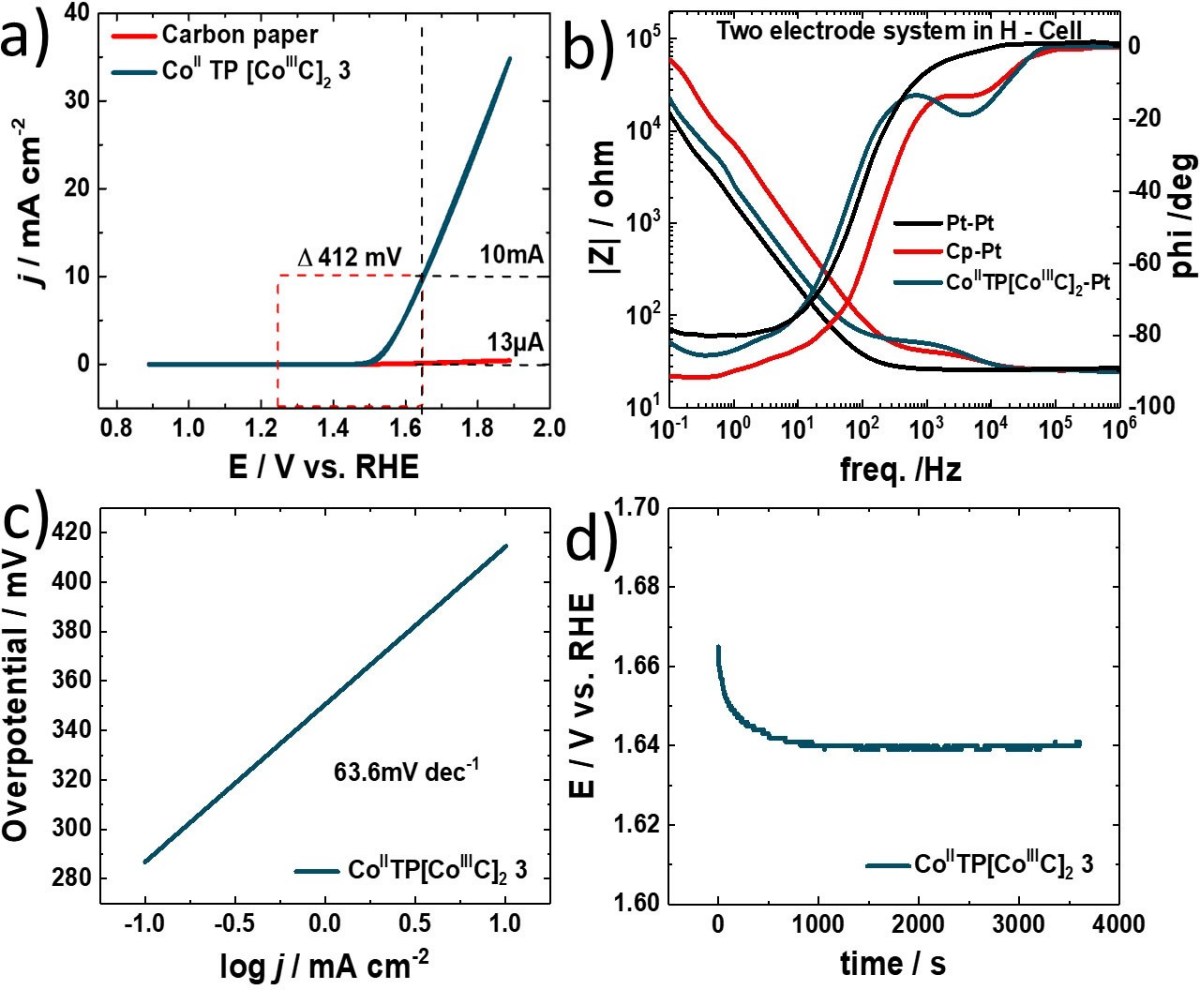
Electrocatalytic OER activity of Co^II^TP[Co^III^C]_2_ **3** electrocatalyst. a) the onset potentials for OER evaluated through LSV measurements of Co^II^TP[Co^III^C]_2_ **3** and Pt. b) Bode plot recorded via electrochemical impedance spectroscopy in the frequency range of 1×10^−1^ Hz to 1×10^6^ Hz with a perturbation amplitude of 10 mV. c) Tafel slopes of the materials in 1 M KOH delivering 63.6 mV dec^−1^ for Co^II^TP[Co^III^C]_2_ **3**. d) chronopotentiometry scan of the Co^II^TP[Co^III^C]_2_ **3** catalyst for 1 hour in 0.1 M KOH at 10 mA.

A proposed mechanism for the oxygen evolution reaction envolves the oxidation of the Co^II^ center to Co^III^ and the axial positions are occupied by water molecules (MALDI‐TOF MS spectra, Figure S18) The water molecule loses a proton and forms the intermediate [(CoC)_2_(TP)_2_(X)Co^III^]^*n*+^ (X=−OH). This species is further oxidized to give the high‐valent cobalt intermediate [(TP)_2_(X)Co^IV^−OH]^*n*+^ which was attacked by a water molecule to form the intermediate [(CoC)_2_(TP)_2_(X)Co^II^−OOH]^*n*+^. The intermediate [(TP)_2_(X)Co^II^−OOH]^*n*+^ was oxidized to form the species [(CoC)_2_(TP)_2_(X)Co^III^−O_2_]^*n*+^ which was further oxidized to release the dioxygen molecule and to generate the initial species [(CoC)_2_(TP)_2_(X)Co^II^−OH_2_]^n+^ for the next catalytic cycle.

To conclude, we report on a highly efficient bifunctional electrocatalyst for ORR and OER. By designing the structure of the catalyst possessing Co^II^ and Co^III^ reaction centers, we achieve nearly 100 % selective activity towards H_2_O_2_ production. The oxygen reduction reaction occurs at the two Co^III^ corrole macrocyclic systems. Promising results are also obtained when applied in OER catalysis. The results indicate relatively low overpotential of 412 mV at current density of 10 mA cm^−2^, Faraday efficiency for oxygen of 98 %, and an outstanding Tafel slope of 64 mV dec^−1^ combined with superior stability. The oxygen evolution reaction occurs at the Co^II^ triazole pyridine subunit of the trimetallic complex. As today's state‐of‐the‐art OER and ORR catalysts mainly rely on expensive noble‐metal based materials and their oxides forms (i.e. Pt, RuO_2_, IrO_2_), an environmentally friendly, efficient, and durable bifunctional alternative is shown for OER and ORR catalysis.

## Conflict of interest

The authors declare no conflict of interest.

## Supporting information

As a service to our authors and readers, this journal provides supporting information supplied by the authors. Such materials are peer reviewed and may be re‐organized for online delivery, but are not copy‐edited or typeset. Technical support issues arising from supporting information (other than missing files) should be addressed to the authors.

Supporting Information

## Data Availability

The data that support the findings of this study are available from the corresponding author upon reasonable request.
